# A case report of drug-induced liver injury after tigecycline administration: histopathological evidence and a probable causality grading as assessed by the updated RUCAM diagnostic scale

**DOI:** 10.1186/s12879-022-07258-w

**Published:** 2022-04-11

**Authors:** Xiaoping Shi, Donghui Lao, Qing Xu, Xiaoyu Li, Qianzhou Lv

**Affiliations:** Department of Pharmacy, Zhongshan Hospital, Fudan University, Shanghai, China

**Keywords:** Tigecycline, Liver injury, Histopathology, Causality assessment, RUCAM

## Abstract

**Background:**

There have been no reports of tigecycline-associated drug-related liver injury (DILI) identified by histopathological assistance and causal assessment method. We reported the histopathological manifestations for the first time and described tigecycline-associated liver injury’s pattern, severity, duration, and outcome.

**Case presentation:**

A 68-year-old male with post-liver transplantation was given high-dose tigecycline intravenously (loading dose 200 mg, followed by 100 mg every 12 h) combined with polymyxin B (50,000 units by aerosol inhalation every 12 h) for hospital-acquired pneumonia caused by carbapenem-resistant *Klebsiella pneumoniae*. At the same time, tacrolimus was discontinued. Liver function was initially normal but started to decline on day 4 of tigecycline. Reducing the dose of tigecycline and resuming tacrolimus could not reverse the deterioration. Therefore, a liver puncture biopsy was performed for further diagnosis, with histopathological findings being cytotoxic injury. The updated RUCAM scale was used to evaluate the causal relationship between tigecycline and liver injury, with the result of 7 points indicating a “probable” causality grading. Methylprednisolone was initiated to treat DILI that was determined to be Grade 1 cholestatic injury. Total bilirubin and transaminase levels returned to normal on day 4 and 11 after tigecycline withdrawal, respectively. Monthly outpatient follow-up showed that the patient’s liver function stayed normal.

**Conclusions:**

This case possessed a significant reference value for differential diagnosis and treatment prognosis of tigecycline-associated DILI. With early diagnosis and timely management, the tigecycline-associated DILI of this patient was successfully reversed.

## Background

Tigecycline is the first glycylcycline antibiotic to be used in clinical practice. Due to its good activity against multidrug-resistant (MDR) bacteria, the use of tigecycline is increasing globally [1]. Despite the gastrointestinal side effects such as nausea and vomiting are more common, it is still worth noting that tigecycline may cause hepatoxicity. A warning was issued by the manufacturer that tigecycline may cause an increase in total bilirubin (TB) and transaminases. The proportion of elevated alanine aminotransferase (ALT) and aspartate aminotransferase (AST) ranged from 6.0 to 55% [2–4]. Guideline of drug-induced liver injury (DILI) defines the lab thresholds criteria for DILI diagnosis after excluding other non-medicinal causes, including ALT exceeding 5-fold of the upper limit of normal (ULN), or ALT over 3-fold of ULN with a 2-fold simultaneous increase in TB [5]. To the best of our knowledge, no tigecycline-associated DILI has been reported. Hereby, we reported a case where the patient developed liver injury after receiving high-dose tigecycline for carbapenem-resistant *Klebsiella pneumoniae* infection. This is the first case where the histopathological manifestations and primary characteristics of liver injury, such as pattern, severity, duration, and outcome, were well described. A comprehensive workup composed of clinical, laboratory, histopathologic investigation was carried out for the final diagnosis of DILI.

## Case presentation

### Liver surgery intensive care unit (LICU) admission and initial anti-infectious therapy

A 68-year-old man with a history of post orthotopic liver transplantation suffered from septic shock in a regular nursing unit. Five hours before transferring to LICU, the patient has had a fever with the highest reading of 41.0 ℃. The patient’s blood pressure dropped to 73/58 mmHg, with heart rate increased to 113 beats/min post transferal. White blood cell (WBC) count was 0.82 × 10^9^/L [reference range 3.50–9.50 × 10^9^/L], and the neutrophil count was 0.0 × 10^9^/L [reference range 1.8–6.3 × 10^9^/L].

Significant past medical history includes chronic hepatitis B virus (HBV) infection for 30 years and hepatocellular carcinoma for 17 months. The patient received his liver transplantation 16 days before the event. After liver transplantation, the HBV DNA was undetectable, while HBsAb, HBeAb, and HBcAb were positive. The patient was taking entecavir 0.5 mg daily. No other known hepatobiliary diseases were found, and the patient denied any history of smoking or alcohol use.

At ICU admission, coarse breath sounds were present in both lungs. The levels of inflammatory markers C-reactive protein (CRP) exceeded 90 mg/L [reference range 0– 10.0 mg/L] and procalcitonin (PCT) was 39.51 ng/mL [reference range < 0.50 ng/mL]. The culture of two sputum samples obtained before LICU admission were positive for MDR *Acinetobacter baumannii*. The patient was started on polymyxin B intravenously of loading dose 1500,000 units with the maintenance of 750,000 units every 12 h, along with meropenem 1 g intravenously every 8 h. The immunosuppressants were discontinued. On day 6 of LICU admission, the culture of the blood sample drawn on day 2 was positive for carbapenem-sensitive *Pseudomonas aeruginosa*, so polymyxin B was stopped. Treated with meropenem and amikacin, the patient’s body temperature and hemodynamic index normalized, and agranulocytosis got corrected. The culture of a follow-up blood sample drawn on day 7 of LICU admission turned negative.

### Occurrence of tigecycline-associated DILI

**Fig. 1:**
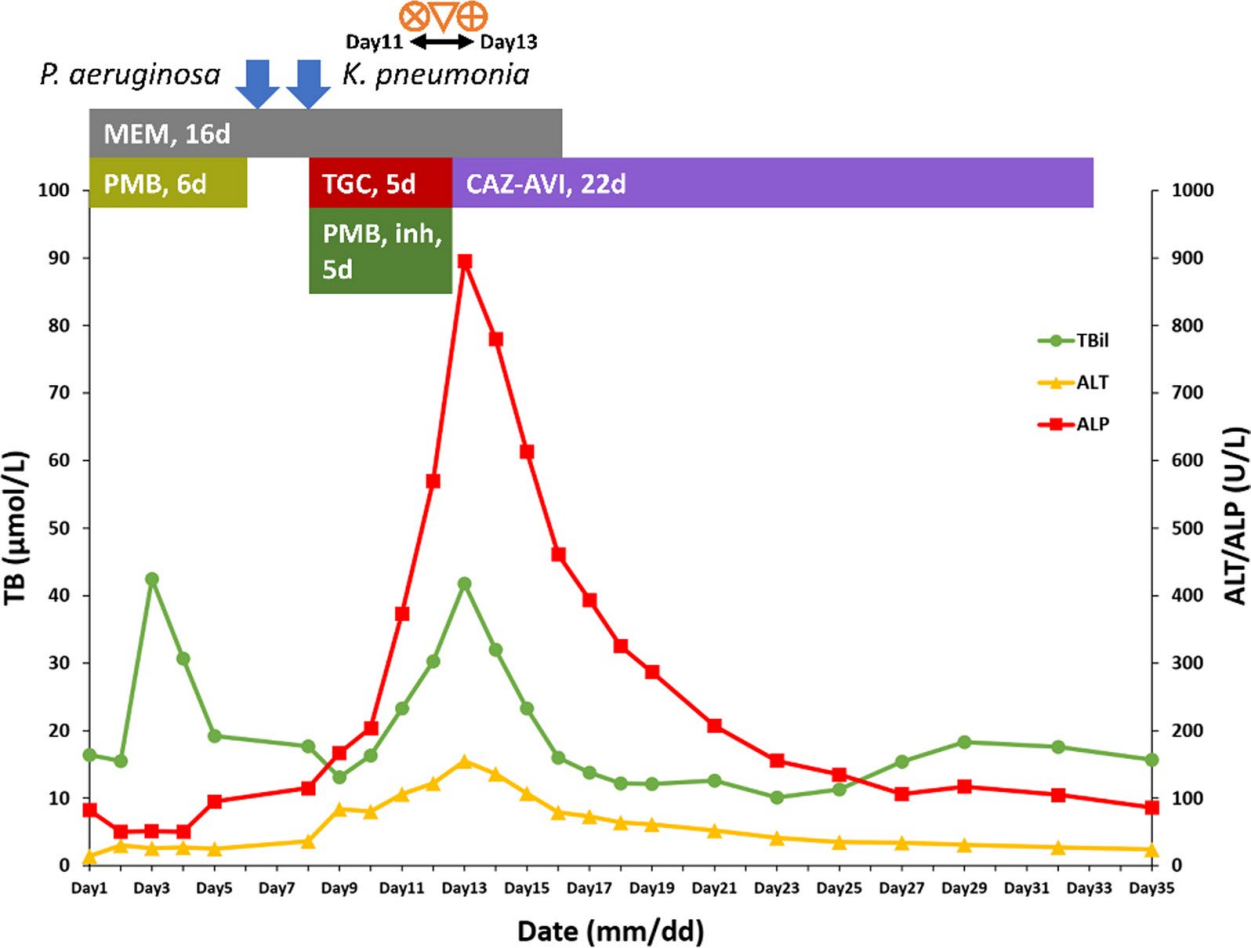
The course, evolution, medication, and events of tigecycline-related DILI. The events of resumption of tacrolimus and reduction of tigecycline dose on day 11 of LICU admission were indicated by  ; The event of liver puncture biopsy on day 12 of LICU admission was indicated by  ; The event of administration of methylprednisolone on day 13 of LICU admission was indicated by  . Abbreviation: TB, total bilirubin; ALT, alanine aminotransferase; ALP, alkaline phosphatase; PMB, polymyxin B; MEM, meropenem; TGC, tigecycline; CAZ-AVI, ceftazidime/avibactam; DILI, drug-induced liver injury

**Fig. 2:**
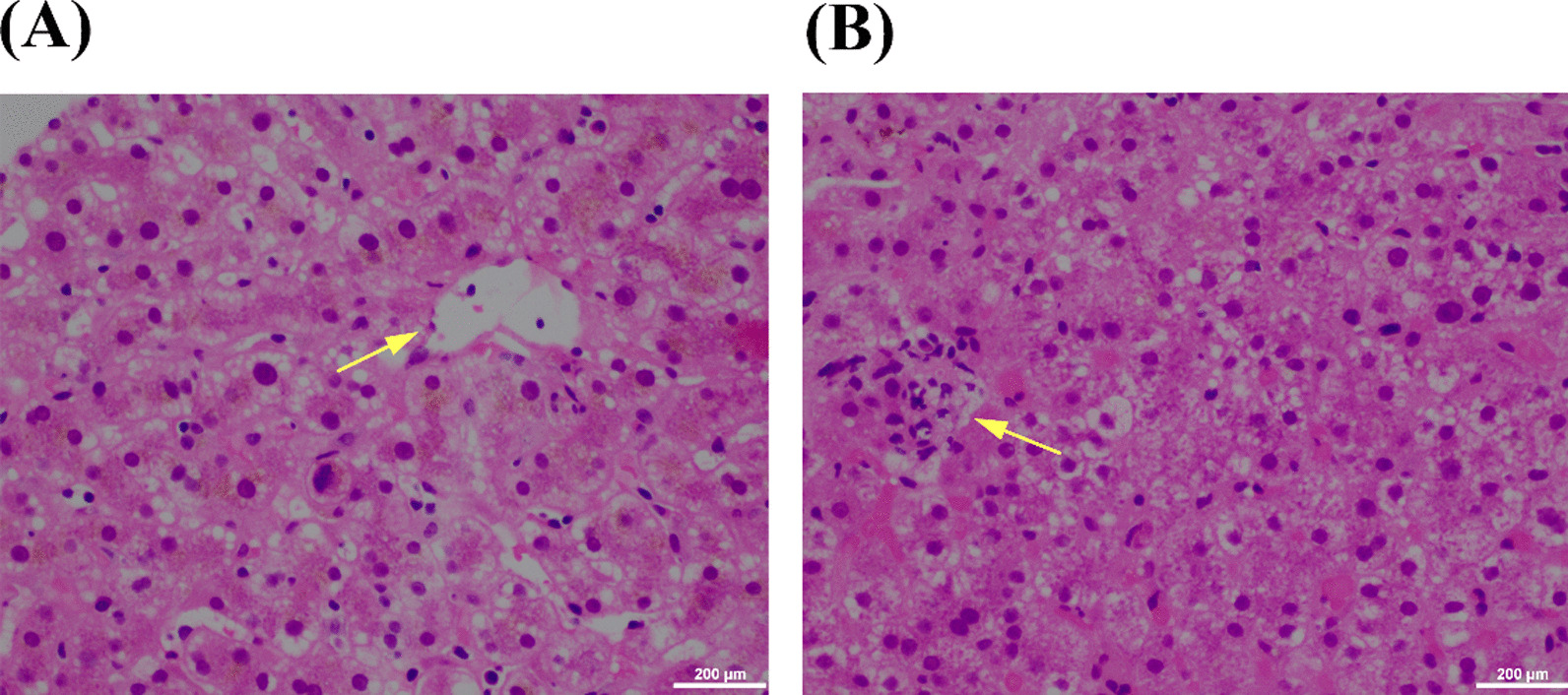
Microscopic features of the liver biopsy specimens. **A** micro cavitation of liver cells, cholestasis (olive green); **B** punctate necrosis. All images were obtained using an OLYMPUS BX43 optical microscope with an attached DP27 digital camera, and CellSens Standard software was used to generate microscopic images

On day 8 of LICU admission, a chest computed tomography (CT) scan showed patchy high-density shadows in the left upper lung and both lower lungs, with stripes and grid-like changes in both lungs. MDR *Klebsiella pneumoniae* was repeatedly detected from the induced sputum. Therefore, the patient was diagnosed with hospital-acquired pneumonia caused by MDR *Klebsiella pneumoniae*. Culture indicated susceptibility to tigecycline, ceftazidime/avibactam (CAZ-AVI), and polymyxin B. Because CAZ-AVI requires the patient’s own expense, high-dose tigecycline (loading dose 200 mg, followed by 100 mg every 12 h intravenously) combined with polymyxin B (50,000 units by aerosol inhalation every 12 h) was used for treatment. However, his liver function started to disorientate, with biochemical levels gradually rising and exceeding the ULN. After four days of intravenous tigecycline, there was a significant increase in the levels of TB, ALT, and alkaline phosphatase (ALP). They increased from 17.7 to 23.3 µmol/L [reference range 3.4–20.4 µmol/L], 36 to 106 U/L [reference range 9–50 U/L], and 115 to 374 U/L [reference range 45–125 U/L], respectively (Fig. 1). A routine post-transplant CT scan of the abdomen was performed three days before liver function declines showed no masses, gallstones, or biliary dilatation. A color Doppler ultrasonography also showed normal blood flow to the transplanted liver. Serological levels of all hepatitis viruses were negative. To prevent further liver injury, immunotherapy of oral tacrolimus 1 mg every 12 h was resumed, with tigecycline dose reduced to 50 mg every 12 h. Nevertheless, the liver function continued to decline. To distinguish whether the reason for liver injury was due to drug factors or liver rejection, we performed a liver puncture biopsy. The histopathological finding was cytotoxic injury. Cholestasis, micro cavitation, and punctate necrosis of liver cells were seen under the microscope (Fig. 2). According to the DILI guideline [5], the updated Roussel Uclaf Causality Assessment Method (RUCAM) scale was used to evaluate the causal relationship between tigecycline and liver injury. The results were 7 points, indicating a “probable” of causality grading. Due to the R ratio being 0.71 [R=(ALT level/ALT ULN)/(ALP level/ALP ULN)], the pattern of tigecycline-associated DILI was determined to be a cholestatic injury with the severity of grade 1.

### Management and outcome of tigecycline-associated DILI

Then, we replaced tigecycline with CAZ-AVI 2.5 g every 8 h intravenously. Methylprednisolone was administered for DILI therapy, with an initial intravenous dose of 80 mg and tapered down to 16 mg orally after 18 days. The trend of the biochemical levels of liver function is shown in Fig. 1. TB levels returned to normal after four days of tigecycline withdrawal, and transaminase levels returned to normal on day 12. The patient was discharged on day 35. A monthly outpatient follow-up lab showed that the patient’s liver function stayed normal post-discharge.

## Discussion and conclusions

DILI diagnosis in liver transplant patients is always challenging for physicians. Apart from the comprehensive differentiation list from other causes, such as rejection, vascular complications, HBV reactivation, and infection, the suspected drug causing DILI is also required to identify various concomitant medications. Failure to notice and diagnose early or manage DILI timely could lead to liver failure and death [6, 7]. The stepwise diagnostic approach for DILI recommended by the European Association for the Study of the Liver (EASL) [5], together with complementary histopathological evidence, provided substantial assistance in diagnosing tigecycline-associated DILI in this case.

Tigecycline is a chemically modified minocycline with increased antimicrobial activity by adding a glycyclamide moiety in the 9-position of minocycline [8]. Minocycline has been reported to be associated with two forms of liver injury, namely acute hepatitis-like syndrome and chronic hepatitis-like syndrome [9–11]. In contrast to minocycline, tigecycline was mainly associated with only transient elevations in transaminases [2–4, 12]. However, the relevant studies did not include patients with severely impaired liver function. Moreover, the liver function biomarkers were not comprehensive, with some studies not defining thresholds criteria for elevated bilirubin or transaminases [2, 3]. This may result in insufficient attention paid to the potential liver hazards of tigecycline. In this case, the diagnosis of DILI for this patient was definite and reliable due to the histopathological evidence and the use of the RUCAM scale recommended by many national and regional guidelines [5, 13, 14]. RUCAM is considered an objective, standardized, and liver-injury-specific approach, which is the most widely used causality assessment tool for DILI diagnosis with the precise definition [15].

Controversies exist for liver injury risk increased by high-dose tigecycline. Two meta-analyses showed no significant difference in the incidence of liver injury in the high-dose group compared with the conventional dose group [16, 17]. However, these two meta-analyses only included 4 to 5 studies when performing subgroup analyses of liver injury. Most of these studies were single-center, retrospective studies with no definition for liver injury provided. Therefore, it is risky to assume no dose-dependent liver injury caused by tigecycline. To achieve optimal exposure for MDR bacteria, we administered tigecycline at an off-label dose [18]. Whether prevailing off-label use of tigecycline will result in more frequent and severe adverse reactions needs to be further studied in the future. We conducted a retrospective study enrolling 1250 patients [19]. The results showed that the incidence of liver injury related to tigecycline was 5.7% and high maintenance dose was one of the independent risk factors. In addition, Dong and her colleagues reported that the risk of liver injury significantly increased when tigecycline trough concentrations above 474.8 ng/mL [20].

At present, the mechanism of DILI caused by tigecycline is still unclear. However, it has been reported that intravenous use of high-dose tetracycline could cause hepatic injury characterized by microvesicular steatosis and lactic acidosis, and liver failure associated with mitochondrial dysfunction and fat metabolism interference in hepatocytes [21]. Tigecycline is a tetracycline derivative. The histopathological manifestations in this patient were similar to a tetracycline-associated liver injury to some extent. Vandecasteele and colleagues reported a case of lethal metabolic acidosis suspected of tigecycline [22]. They suggested that tigecycline could suppress eukaryotic mitochondrial DNA translation. Therefore, we venture to speculate that the mechanism of tigecycline-associated DILI may have similarities with tetracycline-associated DILI.

Long-term use of tigecycline in practice should be avoided. Chen and colleagues’ research suggested that the risk of tigecycline-associated liver injury increases significantly as the administration time increases (beyond 10 days) [2]. The results of our previous study also showed a significant increase in the risk of liver injury with tigecycline treatment over 14 days [19]. Therapeutic drug monitoring can be performed in elderly patients with risk factors for DILI, such as chronic liver disease or diabetes mellitus [5]. The trough concentration of tigecycline is also expected to be lower than 474.8 ng/mL as mentioned above [20].

This is the first article reporting the histopathological manifestations of tigecycline-associated DILI. The pattern, severity, duration, and outcome of liver injury were described in detail, which has significant reference value for differential diagnosis and treatment prognosis. With early detection and timely management, the tigecycline-associated DILI should be reversed.

## Data Availability

The data used and/or analyzed during the current study are available from the corresponding author on reasonable request.

## Abbreviations

DILI: Drug-induced liver injury
RUCAM: Roussel Uclaf Causality Assessment Method
EASL: European Association for the Study of the Liver
LICU: Liver surgery intensive care unit
MDR: Multidrug-resistant
ULN: Upper limit of normal
HBV: Hepatitis B virus
TB: Total bilirubin
ALT: Alanine aminotransferase
AST: Aspartate aminotransferase
ALP: Alkaline phosphatase
WBC: White blood cell
CRP: C-reactive protein
PCT: Procalcitonin
CT: chest computed tomography
CAZ-AVI: ceftazidime/avibactam
